# Does the Effectiveness of Control Measures Depend on the Influenza Pandemic Profile?

**DOI:** 10.1371/journal.pone.0001478

**Published:** 2008-01-23

**Authors:** Solen Kernéis, Rebecca F. Grais, Pierre-Yves Boëlle, Antoine Flahault, Elisabeta Vergu

**Affiliations:** 1 Université Pierre et Marie Curie-Paris6, UMR-S 707, Paris, France; 2 INSERM, UMR-S 707, Paris, France; 3 INRA, UR341 Mathématiques et Informatique Appliquées, Jouy-en-Josas, France; Yale University, United States of America

## Abstract

**Background:**

Although strategies to contain influenza pandemics are well studied, the characterization and the implications of different geographical and temporal diffusion patterns of the pandemic have been given less attention.

**Methodology/Main Findings:**

Using a well-documented metapopulation model incorporating air travel between 52 major world cities, we identified potential influenza pandemic diffusion profiles and examined how the impact of interventions might be affected by this heterogeneity. Clustering methods applied to a set of pandemic simulations, characterized by seven parameters related to the conditions of emergence that were varied following Latin hypercube sampling, were used to identify six pandemic profiles exhibiting different characteristics notably in terms of global burden (from 415 to >160 million of cases) and duration (from 26 to 360 days). A multivariate sensitivity analysis showed that the transmission rate and proportion of susceptibles have a strong impact on the pandemic diffusion. The correlation between interventions and pandemic outcomes were analyzed for two specific profiles: a fast, massive pandemic and a slow building, long-lasting one. In both cases, the date of introduction for five control measures (masks, isolation, prophylactic or therapeutic use of antivirals, vaccination) correlated strongly with pandemic outcomes. Conversely, the coverage and efficacy of these interventions only moderately correlated with pandemic outcomes in the case of a massive pandemic. Pre-pandemic vaccination influenced pandemic outcomes in both profiles, while travel restriction was the only measure without any measurable effect in either.

**Conclusions:**

Our study highlights: (i) the great heterogeneity in possible profiles of a future influenza pandemic; (ii) the value of being well prepared in every country since a pandemic may have heavy consequences wherever and whenever it starts; (iii) the need to quickly implement control measures and even to anticipate pandemic emergence through pre-pandemic vaccination; and (iv) the value of combining all available control measures except perhaps travel restrictions.

## Introduction

The continuous spread of H5N1 avian influenza raises concerns about the possible consequences of a major human influenza pandemic. The three pandemics of the last century each spread differently across the world [1]–[2]. So, although we can learn from past experience, current response plans need to consider the possibility that the eventual pandemic diffusion profile may differ substantially geographically and temporally from previous pandemics.

Mathematical modeling has been used to simulate the spread of a pandemic at a local [3]–[10] and a global scale [11]–[15] and to estimate the impact of different control measures [3]–[19]. Ferguson et al. [3] simulated the spread of a pandemic in South-East Asia and showed that containment at the source was feasible using a combination of antiviral prophylaxis and social distancing measures if the basic reproductive number of the new virus was below 1.8. Longini et al. [4] showed that in the case where interventions were used jointly (targeted antiviral prophylaxis, quarantine and pre-vaccination), the pandemic could be stopped at the source even for basic reproductive numbers as high as 2.4. These results were later extended to the United States and highlighted the potential impact of pre-pandemic vaccination [5]–[6]. Other recent modeling studies have focused on the international spread of an emerging influenza strain taking into account air transportation between countries [11]–[13], [20]. These studies confirm the importance of local control measures and show that restrictions on air travel were unlikely to be of great value in delaying epidemics [11]–[13]. However, the characteristics of a future pandemic could differ substantially from the previous ones. For example, international travel has increased dramatically since the last major pandemic in 1968–1969 and is likely to affect the geographical and temporal spread of the virus.

The great uncertainty on the characteristics of the future influenza pandemic is also due to the uncertainty of key parameters such as the geographical region where the pandemic will start, its season of emergence, the extent of susceptibility of the population to the emerging viral strain, or the epidemiological parameters of influenza like mean durations of latent and infectious periods. Our study aims to identify typical profiles of geographical and temporal diffusion of an influenza pandemic at the global level, taking into account the variability of these parameters. Simulations obtained after sampling the model's parameters were clustered and a multivariate sensitivity analysis was performed to explore how the correlation of different control measures with the pandemic outcomes would vary depending on these profiles. This paper adds to previous work by identifying potential diffusion profiles of a future pandemic on a global scale and by providing new insights on the effectiveness of policies taking into account the great variability in geographical and temporal diffusion.

## Methods

### The Model

The mathematical model used in this study is a refinement of that developed by Flahault et al. [11] and implements a metapopulation approach with coupling between locations through transportation [21]–[22]. The model simulates the spread of a pandemic through a worldwide network of 52 major cities. The epidemic at the city level is simulated by a deterministic model in discrete time, which is composed, when no interventions are modeled, of four compartments representing disease states (Susceptible, Exposed, Infectious, Removed; *S, E, I, R*). Each compartment is divided into five sub-groups corresponding to age groups to which individuals were assigned based on the international population database figures (www.census.gov). The *E* compartment corresponds to the incubation period and individuals become infectious and enter the *I* compartment when symptoms develop. An air traffic matrix connects all cities. This matrix and information on city populations were collected in 2000 by Grais et al. [23]. Individuals in the *I* compartment are supposed to not travel. As the model is formulated in a continuous state space whereas the variables represent discrete quantities (number of individuals), we introduced a control on the number of latent and susceptible individuals similar to Rvachev and Longini [21]: if the sum of all individuals in each compartment at a particular stage is less than one, the compartment is considered empty. This allows the simulation of trajectories leading to extinction as in a stochastic framework, even though this model is deterministic.

The seasonality was accounted through a cosine term in the monthly transmission rate formula:
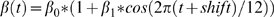
where *β_0_* is the basic transmission rate-defined as the product of the number of contacts per unit of time and the probability of infection given a contact between an infectious and a susceptible individual, in the absence of any seasonality of transmission; *β_1_* is the amplitude of seasonal variation of the basic transmission rate; and *shift* represents the delay in transmission (in months) between Northern and Southern hemispheres. As it is well documented that seasonality of influenza transmission varies with location [24], the 52 cities were classified into one of three distinct regions of seasonal variation of transmission as a good approximation of a more graduated variation: northern and southern zones, characterized by annual cycles in transmission and by a relative delay of 6 months, and tropical regions without any seasonality in transmission.

We also assumed that only a fraction of newly infectious individuals was reported to the authorities.

Six preventive and control measures were integrated into the model: travel restrictions, use of masks, isolation of infectious individuals, antiviral prophylaxis, antiviral therapy and vaccination campaigns (pre-pandemic-with vaccine based on the pre-pandemic strain and pandemic-with vaccine updated for matching pandemic circulating strains).

Input parameters that were varied were divided into two groups: seven parameters related to the pandemic and twenty parameters related to the control measures. Parameters related to the pandemic were: (i) the mean duration of the latent period, (ii) the mean duration of the infectious period, (iii) the city of emergence–characterized by its size, its number of flight connections and the average daily number of travelers from this city (expressed as ordered nominal variables with values representing categories, see Table S2); (iv) the month when the pandemic starts; (v) the basic rate of transmission within the population (*β_0_*); (vi) the amplitude of seasonal effect (*β_1_*); and (vii) the initial proportion of susceptible individuals in the population–assumed to be the same for all cities.

Pandemic vaccination, use of masks, prophylaxis, antiviral therapy and isolation were each characterized by three input parameters: theoretical efficacy, proportion of target population to which the measure is applied, and time lag to introduction (counted from the first case). Pandemic vaccination was also characterized by the duration of the vaccination campaign (time needed to vaccinate target population). Reduction of air traffic was modeled by two parameters: the proportion of air-traffic reduction and the time lag to introduction. Pre-pandemic vaccination was taken into account simply by a coefficient affecting the number of initial susceptible individuals. For antiviral prophylaxis, the theoretical efficacy had two components: one for susceptibility to infection and one for developing the illness if infected.

The effects of vaccination were modelled in our study according to an “all or nothing” action. This means that vaccination confers absolute protection to a given proportion of individuals and no protection to the remaining proportion. Isolation was also taken into account in an “all-or-nothing” manner, and we considered two parameters: the actual proportion of individuals being isolated and the theoretical efficacy of isolation to prevent transmission. In this way, we could take into account possible “leaks” in isolation of ill individuals. Antiviral therapy was considered to reduce the transmission rate of ill patients (illustrating the reduction of infectiousness of those individuals) and also the length of the infectious period by an average of one day [16] (this parameter was not varied in our study).

**Figure 1:**
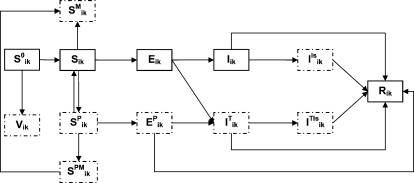
Flow diagram describing the infection spread within a given subgroup k of a city i and the implementation of interventions. At each time, susceptibles (S^0^) could be vaccinated (V) or not (S). The remaining susceptibles could receive prophylaxis (S^P^) during a given time; if not infected at the end of prophylaxis duration they re-enter the susceptible compartment. Susceptibles receiving or not prophylaxis could use masks (S^PM^ and S^M^ respectively, with S^PM^ becoming S^M^ if not infected at the end of antiviral administration period). Once infected, individuals enter the non-infectious latent state (E or E^P^ if under prophylaxis). Infectious symptomatic individuals (I) could be treated (I^T^) (assuming that treatment is administrated in the first day of symptoms, individuals under therapy pass directly from E to I^T^ compartment), isolated (I^Is^) or both (I^TIs^). The R compartment contains all individuals who have been ill and those of latents under prophylaxis that did not develop symptoms.

The model was implemented in Fortran 90: all parameters were specific to each city and to each sub-group, allowing the simulation of a range of eventualities. Figure 1 shows the flow diagram for the epidemic model, describing the different compartments and their interactions for each sub-group (*k*) in each city (*i*). Mathematical details of the model and descriptions of the parameters and values are given in the supplementary information (Appendix S1, Table S1, Table S2 and Table S3).

### Pandemic profiles and impact of control measures

Influenza pandemic profiles and the study of the impact of interventions according to these profiles were identified through several steps:

1. Possible values of input parameters were sampled using the Latin Hypercube Sampling (LHS) method.
2. At first, we sampled values of input parameters related to the characteristics of the pandemic. These values were used to perform 1000 simulations.
3. We applied clustering methods to this set of simulations to identify typical pandemic profiles in the absence of any control measures.
4. A multivariate sensitivity analysis was applied to these 1000 simulations to identify which input parameters had the greatest influence on the temporal and geographical diffusion of the pandemic in the absence of any control measures.
5. In a second step, we focused on two particular pandemic profiles previously identified (at step 3). For each, we performed 1000 simulations using sampled values of input parameters related to the control measures.
6. Next, we performed another multivariate sensitivity analysis to study the independent and relative effects of each control measure on the burden of the pandemic according to the pandemic profile.

### Latin Hypercube Sampling Method

We used the LHS sampling scheme, a type of stratified Monte Carlo sampling first proposed by Mc Kay, Conover and Beckman [25] and later applied to deterministic mathematical models, in particular by Blower et al. [26]. This technique involves several steps: 1) the definition of probability distribution functions for each of the K input parameters; 2) the division of the range of each parameter into N equi-probable intervals; and 3) the generation of the LHS K-sets of parameters by matching at random values sampled without replacement from each probability distribution function.

The ranges of input parameters were taken from previous studies as specified in Table S1. In the absence of available data on the distribution functions, we chose a uniform distribution for all input parameters and large ranges of variation. For more information on the intervals of variation of the input parameters, see Table S1 and Table S2. The proportion of individuals protected by pre-pandemic vaccination was varied between 0 and 0.2 (a range that includes low efficacy scenarios), similar to values considered in a recent work [27]. The theoretical efficacy of the pandemic vaccine (with vaccine strains matching pandemic strains), was considered to be much higher (between 0.3 and 0.7) in agreement with literature values (see Table S1). Similarly, even more restrictive intervals of variation (lower bound = 0.4) were chosen for efficacies of antiviral prophylaxis and therapy.

### Pandemic Profiles

**Figure 2:**
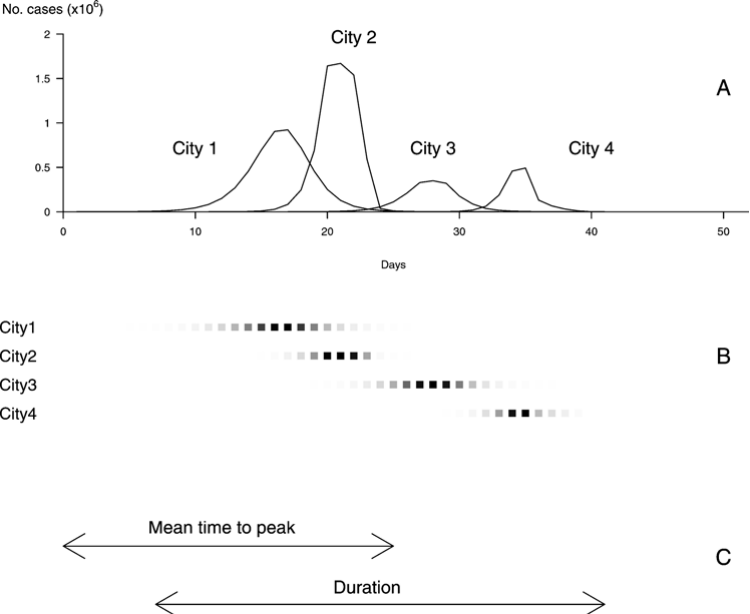
Definition of a pandemic profile and of the outcome variables considered. (A) The upper graph represents the daily incidence of flu in each city affected by the influenza pandemic. The first outcome variable, the cumulated number of cases at the end of the pandemic within all affected cities, is given by sum of areas under the curves of incidence. The second outcome variable, the number of cities affected by the pandemic is given by the number of incidence curves. (B) The day of peak is defined as the day when the incidence rate is maximal. It is represented in each city affected by the pandemic by a deep black square, the level of grey in the other squares being proportional with the daily incidence of flu (scaled separately on the maximum for each city). The cities are represented in the order in which they are affected by the pandemic. (C) The third outcome variable, the mean time to peak, is calculated as the mean time between the start of the pandemic and its peak over all cities affected. The fourth outcome variable represented is the total duration of the pandemic and is defined by the time lag between the apparition of the first case in the first city affected and the last one in the last city. The fifth variable not represented on this graph is the standard deviation of time to peak, calculated as the standard deviation of the time between the start of the pandemic and its peak over all affected cities.

Pandemic profiles were described by five outcome variables: (1) the cumulative number of cases at the end of the pandemic for all affected cities; (2) the total duration of the pandemic defined by the time lag between the first case in the first city affected and the last case in the last city; (3) the number of cities affected by the pandemic; (4) the mean time to peak, calculated as the mean time between the start of the pandemic and its peak over all cities affected; and (5) the standard deviation of the time to peak. The first three outcome variables explored the global burden of the pandemic whereas the last two focused on the dynamics of the pandemic within the network of cities. Figure 2 represents the pandemic's course within four cities of the network, the total duration, the mean time to peak and the total number of cases (the area under the curve of the global incidence). We considered that a city was affected if the daily incidence rate reached 1/100,000. The day of peak was defined as the day when the incidence rate is maximal in each city.

### Clustering methods

Sets of input parameters related to the pandemic sampled using LHS were used in 1000 simulations of the model representing different possible profiles in the absence of any control measure. Typical profiles within the first set of 1000 were identified by hierarchical classification using the Ward's minimum-variance method [28], based on the five outcome variables of the model taken in their standardized form. This is a bottom up method, where objects are iteratively grouped in clusters of increasing size. The algorithm starts with as many clusters as objects, each one containing one object. At each step, the grouping is performed by minimizing the within-cluster sum of squares over all the partitions obtainable by joining two clusters from the previous step. The choice of the number of clusters was based on the values of three criteria: the pseudo t^2^ statistics, the squared multiple correlation *R^2^*-accounting for the proportion of variance explained by the clusters- and the cubic clustering criterion CCC which compares the observed *R^2^* to the expected *R^2^* from a uniform distribution. We considered values of pseudo t^2^ statistics markedly smaller than the consecutive ones (when the number of cluster increases), values of *R^2^* grater than 0.85 and values of CCC greater than 3 indicating a good clustering.

Once the different clusters were identified, a typical profile for the simulated epidemic was determined in each cluster to allow them to be analyzed separately. Since the mean of each cluster was not necessarily a simulated scenario, we selected the trajectory with the minimum sum of squared deviations of the five standardized outcome variables from the cluster mean. The mean of a cluster was defined as the vector of the means of the five output variables. The reproductive rate R in the emerging city at the very beginning of the pandemic was calculated for each profile using a formula that connects it to the rate (*r*) of the exponential increase of an epidemic in its initial phase. We fitted gamma distributions to empirical discrete distributions of latent and infectious durations (*Γ(k_1_,θ_1_)* and *Γ(k_2_,θ_2_)* respectively) and used the corresponding exact expression to compute R [29]: 
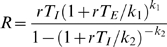
, where T_E_ and T_I_ are the mean duration of the latent and infectious phases respectively.

### Multivariate sensitivity analysis

Two successive multivariate sensitivity analyses were performed, one to identify the input parameters with the greatest influence on the diffusion profile of the pandemic and the other to study the impact of each control measure on each pandemic profile. In both cases, we calculated Partial Rank Correlation Coefficients (PRCCs) between input parameters and output variables. PRCC measures the influence of uncertainty in estimating the values of the input parameter on the imprecision in predicting the value of the output variable [26], [30]. We considered values of PRCC greater than 0.4 as indicating an important correlation between input parameters and output variables and values between 0.2 and 0.4 a moderate correlation.

SAS statistical software (version 9.1) and R statistical package (R Development Core Team; R Foundation for Statistical Computing, Vienna, Austria [http://www.R-project.org]) were used for all statistical analyses.

## Results

### Pandemic profiles

Clustering the first set of 1000 simulations identified six groups of pandemic profiles that could occur in the absence of any control measure.

As reproduced in Table 1, according to the values of clustering criteria, the set of simulated dynamics was split into six subsets, since it performs a significant decreasing in pseudo *t^2^* statistics and corresponds to the first time the 0.85 threshold in *R^2^* values is exceeded.

**Table 1:** Last 10 generations of the clustering history.

Number of clusters	*R^2^*	*t^2^* statistic (PST2)	Cubic Clustering Criterion (CCC)
10	0.915	64.8	35.3
9	0.907	60.4	34
8	0.895	108	31.4
7	0.88	1009	29.1
6*	0.864	50.1	23
5	0.835	258	20.2
4	0.804	86	20.4
3	0.767	70.9	24.6
2	0.543	651	5.65
1	0	1187	0

* The set of simulated dynamics was split into six clusters by considering values of *R^2^* grater than 0.85, values of pseudo *t^2^* statistics markedly smaller than the consecutive ones when reading the table from the bottom and values of CCC greater than 3. The minimum number of clusters satisfying all these criteria is 6.

**Figure 3:**
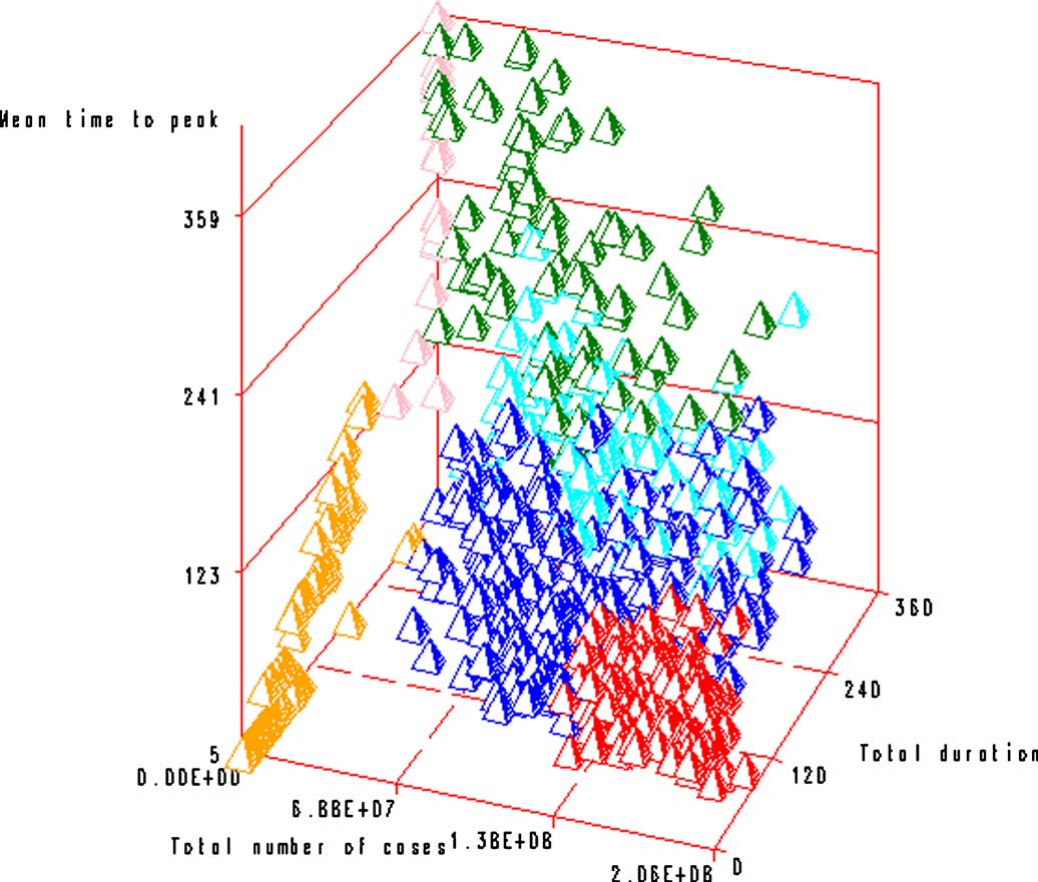
Results of the clustering analysis: the six profiles (profile A in red, B in green, C in blue, D in light blue, E in pink and F in orange) are represented according to three criteria: the total duration, the total number of cases and the mean time to peak.

As is shown in Figure 3, where axes represent three of the discriminating criteria, profiles could be grouped based on (i) the total number of cases: massive pandemics (group A), moderate pandemics (groups B, C and D) and mild pandemics (groups E and F), (ii) duration (groups A and F distinct from groups B, D and E), and (iii) the mean time to peak (groups A and C distinct from groups B and E).

**Figure 4:**
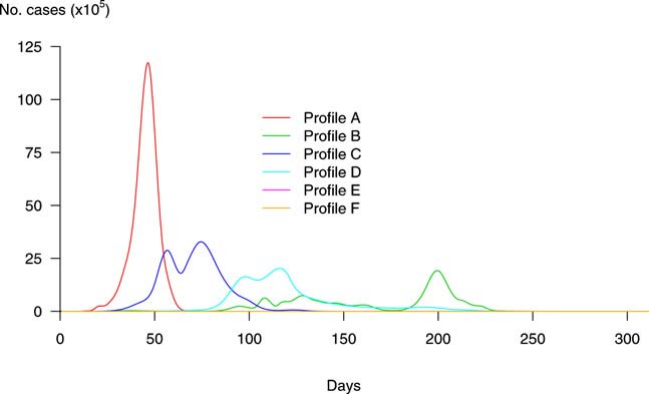
Incidence curves of pandemic profiles identified over 1000 simulated dynamics without control measures. Curve A corresponds to a fast and massive pandemic. Up to 50% of people would be infected worldwide, and all 52 cities of our network would be affected. The global duration would be 89 days and R_A_ = 4.9. Curve B corresponds to a progressive and long lasting pandemic, where 20% of the global population would be infected in 52 cities of our network. The total duration of the pandemic would be around 297 days, and R_B_ = 1.8. Curve E on the x-axis corresponds to a very mild pandemic where cases would represent only 0.1% of the global population. Curve F on the x-axis represents the profile where despite cases in the initial city, the pandemic does not take off. In this case, the number of cases is around 400, which explains why it does not appear clearly on the graph. The other two curves (D and E) show profiles in between, where 35% and 27% respectively of the global population would be attained in 167 and 291 days respectively.

Table 2 contains the characteristics of the six profiles identified as representatives of their respective groups. Figure 4 shows disease incidence over time in the identified profiles in the absence of any control measures.

**Table 2:** Pandemic profiles-corresponding values of input parameters and outcome variables (all time variables are expressed in days).

Profile	City of emergence	Month of emergence	Basic rate of transmission	Amplitude of seasonal effect	Initial % of susceptibles	Mean duration of latent period	Mean duration of infectious period	Total number of cases	Total duration	Mean time to peak	Standard error of peak	Number of cities affected
A	Melbourne	March	1.37	0.69	0.86	1.9	4.0	162×10^6^	89	46	8	52
B	Berlin	March	1.13	0.78	0.39	1.4	3.6	66×10^6^	297	164	44	52
C	Mexico City	January	0.70	0.59	0.75	1.3	3.1	114×10^6^	167	73	15	52
D	Bogota	October	0.65	0.38	0.71	1.4	3.6	86×10^6^	291	128	35	51
E	Manila	September	0.56	0.98	0.44	1.4	2.9	0.32×10^6^	360	200	0	1
F	New York City	March	0.84	0.70	0.29	1.5	3.4	415	26	5	0	1

Profile F corresponds to a situation where, despite initial cases in the city of emergence, the pandemic does not take off. In this case, the number of cases is around 415 (R_F_ = 0.9) in the initial city) and the corresponding incidence curve in Figure 4 is undistinguishable from the x-axis. This scenario with less than 500 cases in only one city is not strictly speaking a pandemic, but rather an influenza outbreak.

**Figure 5:**
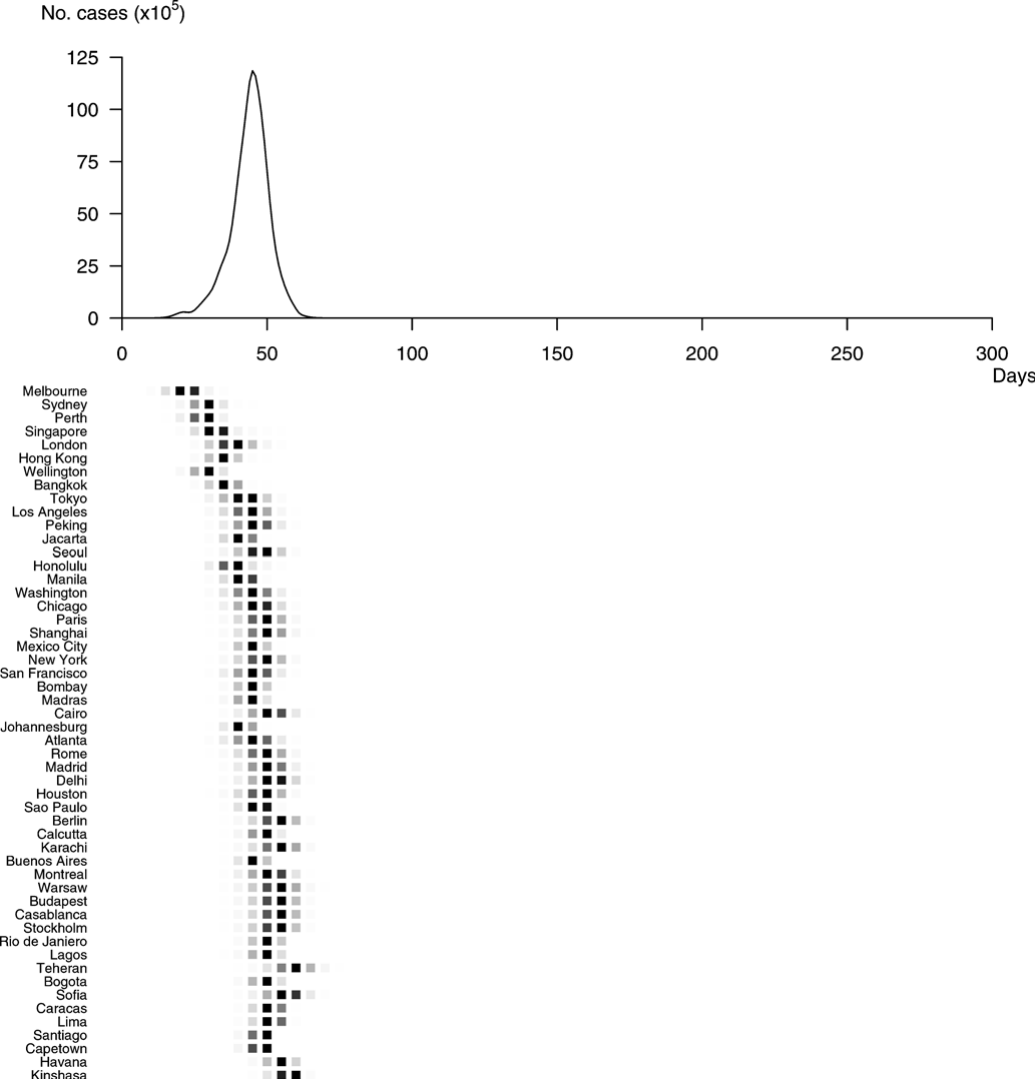
Spatial and temporal spread of Profile A. The pandemic would start in Melbourne and reach all 52 cities of our network. The peak would be reached in all cities affected in less then two months (46 days after the first case). The mean time between peaks in two successively affected cities would be around 8 days. This extreme speed of spread would be associated with a relatively short total duration (89 days).

Profile A corresponds to a rapidly propagating pandemic with high attack rates. The spread of A is detailed in Figure 5. In this case, 86% of individuals are susceptible and the rate of transmission at emergence is 1.37 (R_A_ = 4.9). Up to 50% of people could be identified as infected worldwide, and all 52 cities would be affected. The global incidence would peak 46 days after the first case and the pandemic would spread quickly from one city to another (standard deviation of time to peak = 8 days) with a global duration of 89 days.

**Figure 6:**
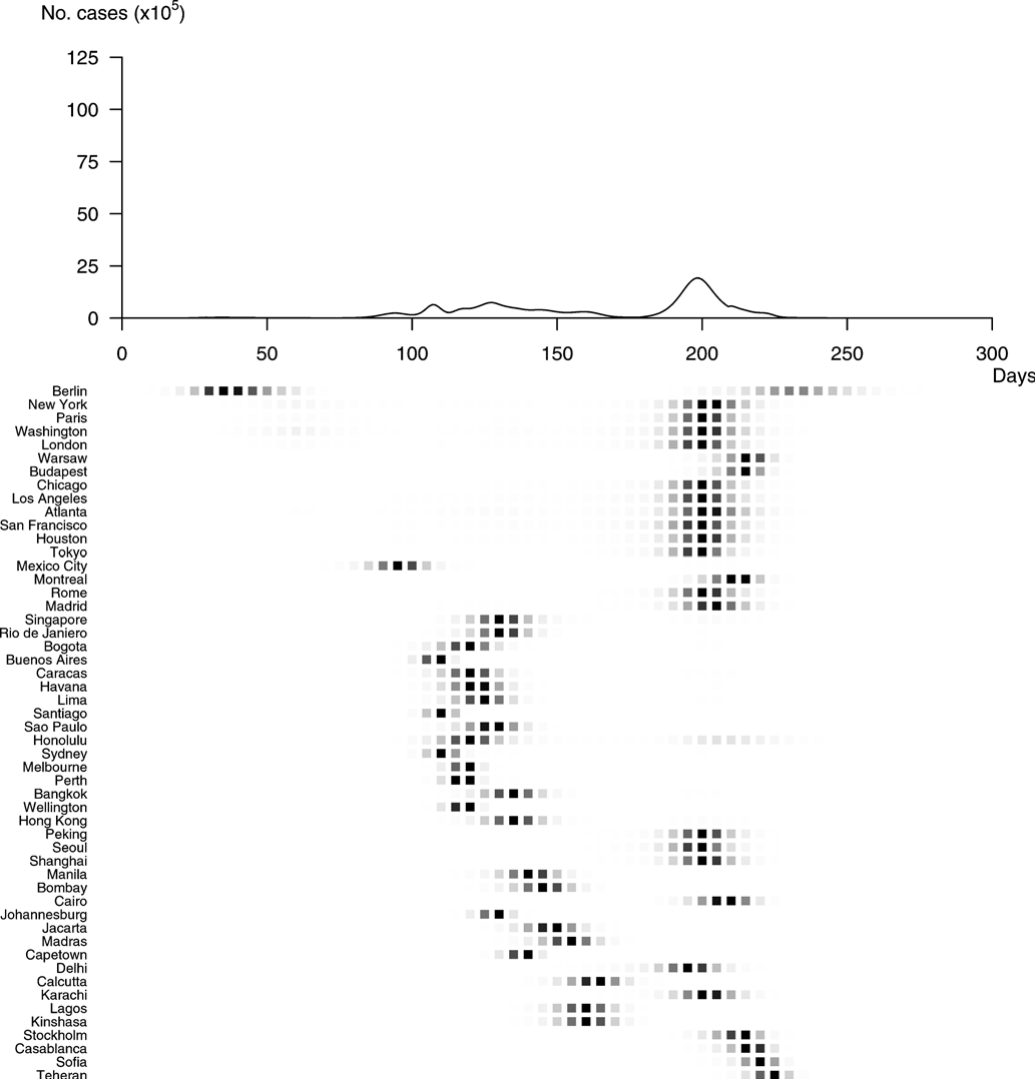
Spatial and temporal spread of Profile B. The pandemic would start in Berlin to reach 52 cities worldwide in a very progressive course. The incidence would peak 164 days after the first case, and the speed of spread would be much lower (standard deviation of time to peak = 44 days). In this scenario, the pandemic would last close to ten months (297 days).

Profile B corresponds to a progressive and long lasting pandemic (Figure 6). In this case, 39% of the global population is susceptible, with a lower rate of transmission at emergence (1.13) and a lower reproductive number (R_B_ = 1.8). Twenty percent of the global population would be reported as infected in all 52 cities. In this scenario, the pandemic wave would spread slowly (standard deviation of time to peak = 44 days) with the peak incidence 164 days after the first case and would last for 297 days.

Profiles C, D and E are in-between these two extremes (represented by profiles A and B) in terms of global burden and total duration (R_C_ = 1.8, R_D_ = 1.6 and R_E_ = 1.1at the source).

### Input parameters influencing the pandemic profile

Table 3 shows the correlations between the input parameters and outcome variables. The basic transmission rate (the rate of transmission in the absence of any seasonality) and the initial proportion of susceptibles correlated most strongly with outcomes. The greatest correlation was between the basic transmission rate and the total number of cases (PRCC = 0.77). The basic transmission rate was also strongly correlated with the number of cities affected (PRCC = 0.70) and moderately associated to the other output criteria. Likewise, the global proportion of susceptibles at the start of the pandemic was strongly correlated with the total number of cases (PRCC = 0.72) and with the number of cities affected (PRCC = 0.50). None of the characteristics of the city of emergence examined (connectivity, population size) correlated with pandemic outcomes. Neither the month of emergence nor the amplitude of the seasonal effect had a significant impact on the spread of the pandemic.

**Table 3:** Absolute values of PRCCs between parameters related to the pandemic and outcome variables.

	Total number of cases	Total duration	Mean time to peak	Standard error of time to peak	Number of cities affected
Mean duration of latent period	0.01	0.07	0.06	0.04	0.04
Mean duration of infectious period	0.04	0.04	0.03	0.04	0.05
City of emergence					
Population size	0.01	0.00	0.01	0.00	0.01
Mean number of connections	0.03	0.03	0.03	0.02	0.02
Mean daily transportation flow	0.00	0.01	0.01	0.01	0.02
Transmission					
Month of emergence	0.04	0.03	0.01	0.03	0.04
Basic rate of transmission	**0.77**	**0.40**	**0.36**	0.41	0.70
Amplitude of seasonal effect	0.16	0.10	0.11	0.03	0.17
Initial proportion of susceptibles*	**0.72**	0.09	0.08	0.16	**0.50**

* The proportion of susceptibles was considered the same in all cities.

### Correlation of control measures with pandemic outcomes

The correlation of interventions with pandemic outcomes was examined in profiles A and B (Figures 5 and 6 respectively). The PRCCs between input parameters and output variables are summarized in Tables 4 and 5, respectively.

**Table 4:** Absolute values of PRCCs between parameters related to the control measures and outcome variables for Profile A corresponding to a fast and massive pandemic (R_A_ = 4.9).

	Total number of cases	Total duration	Mean time to peak	Standard error of time to peak	Number of cities affected
Efficacy					
Pandemic vaccination*	0.03	**0.24**	0.11	0.11	0.03
Masks	0.27	0.28	0.20	0.16	**0.27**
Antiviral Prophylaxis#	0.14/0.08	0.09/0.23	0.09/0.14	0.01/0.11	0.14/0.07
Antiviral Therapy	0.10	**0.27**	0.11	0.12	0.07
Isolation	0.11	**0.20**	0.11	0.08	0.09
Coverage in the target population					
Pandemic vaccination*	0.22	0.33	0.21	0.14	0.18
Masks	0.21	0.29	0.21	0.17	0.21
Antiviral Prophylaxis	0.28	0.56	0.39	0.35	**0.23**
Antiviral Therapy	0.25	0.26	0.12	0.11	0.24
Isolation	0.17	0.37	0.18	0.10	0.15
Pre-pandemic vaccination§	0.30	**0.21**	0.07	0.16	0.27
Proportion of air travel restrictions	0.02	0.02	0.01	0.07	**0.16**
Date of introduction					
Pandemic vaccination	0.28	0.33	0.23	0.17	**0.23**
Masks	0.33	0.15	0.16	0.13	0.32
Antiviral Prophylaxis	**0.76**	**0.58**	**0.69**	**0.50**	0.73
Antiviral Therapy	**0.38**	0.32	0.28	0.31	0.38
Isolation	0.29	0.15	0.12	0.06	0.28
Travel restrictions	0.06	0.05	0.01	0.02	0.20
Duration of the vaccination campaign	0.02	0.01	0.01	0.00	0.01

§ Vaccine composition based on the pre-pandemic strain.

* Vaccine composition updated for matching pandemic circulating strains.

# The first value corresponds to the efficacy for susceptibility to infection and the second one to the efficacy for illness given infection.

**Table 5:** Absolute values of PRCCs between parameters related to the control measures and outcome variables for Profile B corresponding to a long-lasting pandemic (R_B_ = 1.8).

	Total number of cases	Total duration	Mean time to peak	Standard error of time to peak	Number of cities affected
Efficacy					
Pandemic vaccination*	0.01	0.02	0.01	0.00	0.01
Masks	0.04	0.06	0.05	0.03	0.02
Antiviral Prophylaxis#	0.01/0.01	0.03/0.04	0.04/0.03	0.05/0.03	0.01/0.02
Antiviral Therapy	0.02	0.05	0.02	0.04	0.03
Isolation	0.04	0.07	0.02	0.06	0.01
Coverage in the target population					
Pandemic vaccination*	0.07	0.16	0.09	0.07	0.06
Masks	0.09	0.10	0.09	0.07	0.07
Antiviral Prophylaxis	0.06	0.09	0.06	0.03	0.07
Antiviral Therapy	0.06	0.12	0.08	0.11	0.06
Isolation	0.07	0.14	0.10	0.09	0.04
Pre-pandemic vaccination§	0.48	0.16	0.01	0.31	0.43
Proportion of air travel restrictions	0.03	0.06	0.06	0.05	0.09
Date of introduction					
Pandemic vaccination	0.28	0.42	0.32	0.25	0.23
Masks	0.21	0.19	0.23	0.16	0.19
Antiviral Prophylaxis	0.30	0.44	0.34	0.29	0.24
Antiviral Therapy	0.29	0.31	0.30	0.23	0.26
Isolation	0.19	0.26	0.22	0.14	0.15
Travel restrictions	0.03	0.04	0.08	0.04	0.14
Duration of the vaccination campaign	0.05	0.07	0.09	0.08	0.06

§ Vaccine composition based on the pre-pandemic strain.

* Vaccine composition updated for matching pandemic circulating strains.

# The first value corresponds to the efficacy for susceptibility to infection and the second one to the efficacy for illness given infection.

Regardless of the profile, restricting air travel (either expressed by the proportion and the date of introduction of transport limitation) had no impact on the global burden of the pandemic. Only the date at which travel restrictions are introduced correlated slightly with the number of cities affected (profile A, PRCC = 0.20; profile B PRCC = 0.14).

The other main finding is that early introduction of other control measures is the most important factor to reduce the number of infections, regardless of the profile and for all interventions considered. In profile A, it impacted mainly on the number of cases, the number of cities affected and the duration (PRCCs ranging from 0.28 to 0.76, from 0.23 to 0.73 and from 0.15 to 0.58 respectively), and other outcomes also showed important correlation. In profile B, date of introduction of control measures (again excepting travel limitation) correlated slightly less with outcomes and in a more homogeneous manner (PRCCs for all output variables in the range 0.14–0.44).

Apart from air traffic reductions, the effectiveness of control measures varied depending on the pandemic profile. In case of a fast and massive pandemic (profile A), efficacy and coverage play a moderate role for several interventions, whereas in a progressive and long lasting pandemic (profile B), such correlations do not clearly appear, except for speed of intervention (as mentioned above) and pre-pandemic vaccination. In this case, PRCCs show moderate correlation between efficacy of pre-pandemic vaccine and total number of cases, standard error of time to peak, and number of cities affected (PRCC = 0.48, 0.31 and 0.43 respectively). Profile B is characterized by a weak correlation between the proportion of individuals being vaccinated with the pandemic strain, using masks, or being treated or isolated and the total duration of the pandemic (PRCC from 0.10–0.16).

For profile A, the PRCC of the efficacy for all interventions is higher than 0.10 for at least one of the outcome variables. In terms of theoretical efficacies, the interventions having an impact on the pandemic dynamics are masks (PRCC>0.25 for the total number of cases, the duration and the number of cities affected), antiviral therapy (PRCC = 0.10 and 0.27 for total number of cases and duration, respectively), pandemic vaccination (PRCC = 0.24 for the total duration) and isolation (PRCC = 0.11 and 0.20 for total number of cases and duration, respectively).

The proportions of individuals of target populations to which interventions are applied are also correlated with outcomes: the coverage of prophylaxis have the greatest impact on all criteria (PRCCs between 0.23 and 0.56), but coverage of pandemic vaccination, antiviral therapy, masks use and isolation also influence the pandemic dynamics (PRCC of respectively 0.33, 0.26, 0.29 and 0.37 with the total duration). Profile A is also characterized by moderate correlations between the global effect of pre-pandemic vaccination and the total number of cases and of cities affected (PRCC equal to 0.30 and 0.27 respectively).

From the point of view of the output variables, the global pandemic burden and the total duration seem to concentrate the most of the impact of input parameters. However, this pattern is less obvious for the profile B.

## Discussion

Using a mathematical model, we identified six typical profiles of geographical and temporal spread of an influenza pandemic, and the two key parameters influencing these profiles: the proportion of susceptible individuals in the initial population and the basic rate of transmission between individuals. Supplementary analyses performed separately on each of two selected profiles suggest that the variation in the impact of pandemic control measures and the spatial-temporal pattern subsequent to their implementation depend on the pandemic profile.

Although not unexpected, the importance of the proportion of susceptible individuals in the population may have important policy implications. The fact that not all individuals are susceptible to the pandemic strain represents cross-immunity with previously circulating viruses. This assumption is supported, for instance, by what was observed during the 1968/A/H3N2 pandemic in United States: a reduced mortality burden with respect to that of the previous pandemic which occurred in 1957 and was also caused by an A/H3N2 strain. One possible explanation is that human population was partially protected in 1968 against H3N2 strain due to antibodies to N2 allele acquired after the 1957 pandemic [31]. In large urban areas or mega-cities, the pandemic virus will continue to spread even if only a small proportion of the population is susceptible, but it will not in less populated areas. Where resources are potentially limited, these results stress the importance of focusing control efforts on densely populated areas. Targeting high transmitters such as children would be an equally important step to limit transmission, since transmission rate was also identified as being strongly correlated with pandemic outputs.

The central role of the proportion of susceptibles also indirectly illustrates the potential benefits of pre-pandemic vaccination, which aims to reduce the susceptibility of individuals before the emergence of the pandemic strain. It is therefore not surprising that in this model, pre-pandemic vaccination correlates with the number of cases whatever the pandemic profile. Although the efficacy of pre-pandemic vaccine remains uncertain, pre-pandemic vaccination should still be useful even at a low level of efficacy [27]. As our simulation results suggest, it could be beneficial if, on average, complete protection is conferred to at least a proportion of population ranging from 0 to 0.2. In addition, for a given duration of infection and a specific transmission rate, there is a minimum threshold of susceptible individuals in the city of emergence required for virus propagation, and hence the spread of the pandemic itself. As illustrated by profile F, with a basic rate of transmission of 0.84 and 29% of the population susceptible in the city of emergence (much lower than the required threshold) only one city and 415 individuals were affected. Any interventions which might lower the number of susceptible individuals below this theoretical threshold might go a great way to preventing a pandemic.

It is also noteworthy that the city of emergence, the month of emergence and seasonality do not play a major role in the profile of a pandemic. According to field evidence, it seems that pandemic flu is more likely to start in a region where there is close proximity between humans and their poultry, a point that was not explicitly included in our modelling approach. However, our simulation analysis shows that a pandemic is likely to occur independently of the characteristics of the city of origin, like its size or its number of air connections. Since the best way to mitigate its consequences is to contain it at source [3]–[4] this highlights the importance of having every country as prepared as possible to react quickly if the pandemic emerges on its soil.

The variation of mean duration of latent and infectious periods also did not result in significant PRCC values with any of outcome variables. This finding, a little surprising at a first glance, could have at least two explanations: 1) the relatively small range of variation (between 1.2 and 1.9 for T_E_ and from 2.5 to 4, for T_I_, where these values were taken from the literature [3]–[4]); and 2) the relatively weak impact of these parameters' variation in relation, for instance, to the initial proportion of susceptibles, in the frame of a multivariate sensitivity analysis. This last point is supported by important correlations coefficients between T_E_ and T_I_ with the total number of cases (0.93 and 0.88 respectively, data not shown) but weak relative variation of the global burden (factors of 1.04 and 1.38 when bounds of variation intervals are considered for T_E_ and T_I_ respectively) computed in the case of a univariate analysis.

Our results also suggest that travel restrictions would have a limited impact on the spatial and temporal diffusion of an influenza pandemic. Indeed, regardless of the pandemic profile, restricting air travel in our model has little effect on the global burden of the pandemic. Such restrictions have significant logistical, ethical and economic implications and their impact on an influenza pandemic is currently debated [6], [9], [12], [13], [20], [23], [32].

Our research also highlights the importance of a timely response. Regardless of the spatial-temporal profile, the timing of interventions is crucial, underlining the need for vigilant and sensitive surveillance to ensure an early detection and timely response. It also stresses the added value of pre-pandemic vaccination, which can be used immediately, even if less efficacious than the appropriate pandemic vaccine which may take several months to be produced and distributed. But at this stage, it is impossible to predict which proportion of susceptibles will actually be immunized by a vaccine based on a pre-pandemic strain, and the effectiveness of this control measure is strongly correlated with this missing information. The choice of using such pre-pandemic vaccine should probably rely on preliminary immunogenicity studies.

The date of introduction of most of the control measures considered correlated with pandemic outcomes whatever the pandemic profile, although coverage and theoretic efficacy were more strongly correlated to the outcomes of a fast, massive pandemic than a long-lasting pandemic. This can be interpreted as the need for a control measure to be used at a very large scale to have a real impact in the case of a massive pandemic. This supports the idea that that a very aggressive pandemic will be very difficult to mitigate given the constraints on resource availability [5]–[6]. Conversely, this result stresses the value of measures not relying on stockpiled resources such as isolation [18], measure that correlated moderately by its coverage with the total duration in the case of a massive pandemic. Regardless of the profile, the date of isolation introduction also correlated with outcomes. When evaluating the potential impact of isolation measures, one should have in mind that their outcome could be influenced by the pre-symptomatic or asymptomatic individuals, as it was discussed in Fraser et al. [33]. Here, we assumed that only infectious symptomatic individuals who become infectious at the end of incubation period transmit. According to experimental and observational studies, viral shedding arises at low levels a short while before the onset of symptoms [34]. However, the public health impact of pre-symptomatic transmitters still remains unclear and could not be quantified precisely since there are few field studies reporting infections from such infected individuals [35]. Nevertheless, considering the potential importance of such transmitters on the outcome of isolation-like interventions, we consider this statement in an indirect manner by assuming that isolation efficacy could not be greater than 70%.

When interpreting the results of this analysis, it must be remembered that most are expressed in terms of correlation with outcomes and not in terms of level of impact. Our correlation results express the ability to improve the results each time a control measure is more (or less) extensively used (rank correlation). A low correlation coefficient does not necessarily mean an absence of impact. It means that increasing the use of a control measure is not systematically beneficial.

Beyond the results for any one specific measure, our analysis highlights the value for every country looking to limit the potential devastating consequences of a pandemic to 1) not rely on a single control measure but use them all to complement each other, 2) be prepared with response planning, and stockpiling of antivirals and vaccines and 3) monitor the progression of the pandemic and adapt the response to its profile.

The general applicability of our conclusion may be limited by the following considerations. Firstly, we used air travel data from 2000 and for 52 global cities. Although updating air travel data and including more cities in the model might improve its accuracy, these values were chosen to be representative of global air travel volume and world geography. Secondly, we used a deterministic, discrete time formula that has been shown to be suitable for use in large populations. Since the dynamics of internal epidemics within cities was not the focus of this research, but rather the global spread, this type of approach would seem appropriate. Nevertheless, since we extensively explored the model behaviour by performing multivariate sensitivity, we can be confident that our modelling approach reproduced a number of realistic potential scenarios and provides, in this sense, a panel of pandemic dynamics analogue to a fully stochastic model. The fact that our analyses led to similar conclusions to previous studies using a slightly different methodology does not make them realistic, but points to probable robustness of these conclusions.

In conclusion, our key finding concerning the dependence of the efficiency of interventions on the pandemic profile demonstrates the critical importance of developing tools for early-stage identification of the pandemic profile in order to adapt the public health response in as timely a manner as possible.

## Supporting Information

Table S1 Probability distribution functions and ranges of values for input parameters that were made vary in order to carry out sensitivity analyses.
(0.02 MB PDF)

Table S2 Characteristics of the initial city: ranges of values and the corresponding categories.
(0.05 MB PDF)

Table S3 Input parameters that do not vary over time and their respective values.
(0.08 MB PDF)

Appendix S1 Technical appendix
(0.18 MB PDF)
