# Supplementary material for: Does the Effectiveness of Control Measures Depend on the Influenza Pandemic Profile?
Source: PLoS One. 2008 Jan 23;3(1):e1478. doi: 10.1371/journal.pone.0001478 (PMC2198944; doi:10.1371/journal.pone.0001478)
Supplement: Table S1 — Probability distribution functions and ranges of values for input parameters that were made vary in order to carry out sensitivity analyses. (0.02 MB PDF) [file pone.0001478.s001.pdf]

Table S1: Probability distribution functions and ranges of values for input parameters that were made vary in order to carry out sensitivity analyses.

Parameter	Probability distribution functions			References
	Min	Max	Function shape	
Input parameters related to the pandemic				
Mean duration of latent period (in days)	1.2	1.9	10 distinct values between min and max were tested	[1,2]
Mean duration of infectious period (in days)	2.5	4.0	10 distinct values between min and max were tested	[1,2]
City of emergence	1	52	Discrete Uniform	
Month of emergence	1	12	Discrete Uniform	
Basic rate of transmission	0.01	1.5	Uniform continuous	[3-6]
Amplitude of seasonal effect	0.1	1	Uniform continuous	
Initial proportion of susceptibles	0.25	1	Uniform continuous	[7-12]
Input parameters related to interventions				
Efficacy of the pandemic vaccine	0.3	0.7	Uniform continuous	[5, 13-17]
Efficacy of masks for susceptibility to infection	0	1	Uniform continuous	
Efficacy of antiviral prophylaxis for susceptibility to infection	0.2	0.5	Uniform continuous	[1,18-19]
Efficacy of antiviral prophylaxis for illness given infection	0.4	0.8	Uniform continuous	[1]
Efficacy of antiviral therapy for infectiousness	0.4	0.8	Uniform continuous	[1,18,20]
Efficacy of isolation	0.2	0.7	Uniform continuous	[21]
Proportion of individuals to which the measure is applied (coverage in the target population)				
Pandemic vaccination (initial susceptibles*)	0.001	1	Uniform continuous	
Masks (susceptibles non vaccinated*)	0.001	1	Uniform continuous	
Antiviral Prophylaxis (susceptibles non vaccinated*)	0.001	1	Uniform continuous	
Antiviral Therapy (ill*)	0.001	1	Uniform continuous	
Isolation (ill, treated or non treated*)	0.001	1	Uniform continuous	
Proportion of susceptible individuals completely protected after the pre-pandemic vaccination campaign	0	0.2	Uniform continuous	[22]
Reduction of air traffic	0.001	1	Uniform continuous	
Date of introduction (in days)				
Pandemic vaccination	5	60/250**	Discrete Uniform	
Masks	5	60/250**	Discrete Uniform	
Antiviral Prophylaxis	5	60/250**	Discrete Uniform	
Antiviral Therapy	5	60/250**	Discrete Uniform	
Isolation	5	60/250**	Discrete Uniform	
Travel restrictions	5	60/250**	Discrete Uniform	
Duration of per-pandemic vaccination campaign (in days)	10	30	Discrete Uniform	

*Target population (representing the denominator when calculating the proportion of individuals to whom the intervention is applied) is indicated in brackets;

**For the date of interventions introduction, the upper bound was different according to the profile that was analyzed: 60 corresponds to the short and massive pandemic lasting 89 days and 250 to the long-lasting pandemic profile lasting 297 days.

An extensive literature research aimed at collecting information on the intervals of variation of each input parameter and on the possible shape of the probability distribution functions. When no information was available on the limits of the interval, we defined an interval of variation as large as possible to explore all possible values of the input parameter.
In the absence of precise information on the shapes of the probability distribution functions, we have chosen Uniform distributions for all input parameters and large ranges of variation.

References

1. Longini IM, Jr., Nizam A, Xu S, Ungchusak K, Hanshaoworakul W, et al. (2005) Containing pandemic influenza at the source. Science 309:1083-1087.
2. Ferguson NM, Cummings DA, Cauchemez S, Fraser C, Riley S, et al. (2005) Strategies for containing an emerging influenza pandemic in Southeast Asia. Nature 437:209-214.
3. Mills CE, Robins JM, Lipsitch M (2004) Transmissibility of 1918 pandemic influenza. Nature 432:904-906.
4. Longini IM, Jr., Halloran ME, Nizam A, Yang Y (2004) Containing pandemic influenza with antiviral agents. Am J Epidemiol 159:623-633.
5. Patel R, Longini IM, Jr., Halloran ME (2005) Finding optimal vaccination strategies for pandemic influenza using genetic algorithms. J Theor Biol 234:201-212.
6. Flahault A, Letrait S, Blin P, Hazout S, Menares J, et al. (1988) Modelling the 1985 influenza epidemic in France. Stat Med 7:1147-1155.
7. Flahault A, Deguen S, Valleron AJ (1994) A mathematical model for the European spread of influenza. Eur J Epidemiol 10:471-474.
8. Rvachev L, Longini IM, Jr. (1985) A mathematical model for the global spread of influenza. Math Biosci 75:3-22.
9. Grais RF, Ellis JH, Kress A, Glass GE (2004) Modeling the spread of annual influenza epidemics in the U.S.: the potential role of air travel. Health Care Manag Sci 7:127-134.
10. Cliff AD, Haggett P (1993) Statistical modelling of measles and influenza outbreaks. Stat Methods Med Res 2:43-73.
11. Cauchemez S, Carrat F, Viboud C, Valleron AJ, Boelle PY (2004) A Bayesian MCMC approach to study transmission of influenza: application to household longitudinal data. Stat Med 23:3469-3487.
12. Stilianakis NI, Perelson AS, Hayden FG (1998) Emergence of drug resistance during an influenza epidemic: insights from a mathematical model. J Infect Dis 177:863-873.
13. Bridges CB, Thompson WW, Meltzer MI, Reeve GR, Talamonti WJ, et al. (2000) Effectiveness and cost-benefit of influenza vaccination of healthy working adults: A randomized controlled trial. Jama 284:1655-63.
14. Zangwill KM, Belshe RB (2004) Safety and efficacy of trivalent inactivated influenza vaccine in young children: a summary for the new era of routine vaccination. Pediatr Infect Dis J 23:189-197.
15. Germann TC, Kadau K, Longini IM, Jr., Macken CA (2006) Mitigation strategies for pandemic influenza in the United States. Proc Natl Acad Sci U S A 103:5935-5940.

16. Longini IM, Halloran ME, Nizam A, Wolff M, Mendelman PM, et al. (2000) Estimation of the efficacy of live, attenuated influenza vaccine from a two-year, multi-center vaccine trial: implications for influenza epidemic control. Vaccine 18:1902-1909.
17. Longini IM, Jr., Halloran ME (2005) Strategy for distribution of influenza vaccine to high-risk groups and children. Am J Epidemiol 161:303-306.
18. Hayden FG (2001) Perspectives on antiviral use during pandemic influenza. Philos Trans R Soc Lond B Biol Sci 356:1877-1884.
19. Welliver R, Monto AS, Carewicz O, Schatteman E, Hassman M, et al. (2001) Effectiveness of oseltamivir in preventing influenza in household contacts: a randomized controlled trial. Jama 285:748-754.
20. Ferguson NM, Mallett S, Jackson H, Roberts N, Ward P (2003) A population-dynamic model for evaluating the potential spread of drug-resistant influenza virus infections during community-based use of antivirals. J Antimicrob Chemother 51:977-990.
21. Nuno M, Chowell G, Gumel AB (2007) Assessing the role of basic control measures, antivirals and vaccine in curtailing pandemic influenza: scenarios for the US, UK and the Netherlands. J R Soc Interface 4:505-521.
22. Riley S, Wu JT, Leung GM (2007) Optimizing the dose of pre-pandemic influenza vaccines to reduce the infection attack rate. Plos Med 4:e218.
