# Supplementary material for: Does the Effectiveness of Control Measures Depend on the Influenza Pandemic Profile?
Source: PLoS One. 2008 Jan 23;3(1):e1478. doi: 10.1371/journal.pone.0001478 (PMC2198944; doi:10.1371/journal.pone.0001478)
Supplement: Table S2 — Characteristics of the initial city: ranges of values and the corresponding categories. (0.05 MB PDF) [file pone.0001478.s002.pdf]

Table S2: Characteristics of the initial city: ranges of values and the corresponding categories.

	Category 1	Category 2	Category 3	Category 4	Category 5	Category 6
Population size (x 10^3)	≤ 2400	2401 –3500	3501 –5000	5001 – 9000	9001 – 13000	13001 –24000
Mean daily flow	≤ 2000	2001 – 5000	5001 – 8000	8001 –19000	20001 –30000	30001 –70000
Mean number of connections	≤ 19	20 – 29	30 – 39	40 – 49	> 49	-

For each city, the following variables have been considered: population size, mean daily flow of individuals traveling from this city and mean number of connections between this city and the other cities of our network. In our analyses, these variables have been categorized as presented in the table.
