# Supplementary material for: Does the Effectiveness of Control Measures Depend on the Influenza Pandemic Profile?
Source: PLoS One. 2008 Jan 23;3(1):e1478. doi: 10.1371/journal.pone.0001478 (PMC2198944; doi:10.1371/journal.pone.0001478)
Supplement: Table S3 — Input parameters that do not vary over time and their respective values. (0.08 MB PDF) [file pone.0001478.s003.pdf]

Table S3: Input parameters that do not vary over time and their respective values.

Input parameters related to the pandemic	Value	References
Maximum day of latent period	2 days	[1,2]
Maximum day of infectious period	8 days	[1,2]
Reporting rate of cases to authorities	0.6	[3-5]
Number of cities	52	
Seed (i.e. number of individuals of initial city in latent state at the beginning of the pandemic)	10^{-5} of the population size of the initial city	[1]
Temporal shift in transmission between Northern and Southern hemispheres	6 months	[2,4,6,7]
**Input parameters related to interventions**		
Duration of prophylaxis for each individual	10 days	
Mean duration of infectious period under antiviral therapy	(T_I* – 1) days	[3,4,8]
Time lag between the first case in the initial city and the start of air traffic between cities	5 days	

* T_I: Mean duration of the infectious period in the absence of any intervention.

References

1. Rvachev L, Longini IM, Jr. (1985) A mathematical model for the global spread of influenza. Math Biosci 75:3-22.
2. Grais RF, Ellis JH, Kress A, Glass GE (2004) Modeling the spread of annual influenza epidemics in the U.S.: the potential role of air travel. Health Care Manag Sci 7:127-134.
3. Germann TC, Kadau K, Longini IM Jr, Macken CA (2006) Mitigation strategies for pandemic influenza in the United States. Proc Natl Acad Sci USA 103:5935-5940.
4. Ferguson NM, Mallett S, Jackson H, Roberts N, Ward P (2003) A population-dynamic model for evaluating the potential spread of drug-resistant influenza virus infections during community-based use of antivirals. J Antimicrob Chemother 51:977-990.
5. Stilianakis NI, Perelson AS, Hayden FG (1998) Emergence of drug resistance during an influenza epidemic: insights from a mathematical model. J Infect Dis 177:863-873.
6. Cox NJ, Subbarao K (2000) Global epidemiology of influenza: past and present. Annu Rev Med 51:407–421.
7. Dushoff J, Plotkin JB, Levin SA, Earn DJ (2004) Dynamical resonance can account for seasonality of influenza epidemics. Proc Natl Acad Sci USA 101:16915-19916.
8. Longini IM, Jr., Halloran ME, Nizam A, Yang Y (2004) Containing pandemic influenza with antiviral agents. Am J Epidemiol 159:623-633.
