# Supplementary material for: Does the Effectiveness of Control Measures Depend on the Influenza Pandemic Profile?
Source: PLoS One. 2008 Jan 23;3(1):e1478. doi: 10.1371/journal.pone.0001478 (PMC2198944; doi:10.1371/journal.pone.0001478)
Supplement: Appendix S1 — Technical appendix (0.18 MB PDF) [file pone.0001478.s004.pdf]

Does intervention effectiveness depend on the influenza pandemic profile?

Technical appendix

Solen Kernéis[1,2], Rebecca F. Grais[1,2], Pierre-Yves Boëlle[1,2], Antoine Flahault[1,2], Elisabeta Vergu[3]

[1]Université Pierre et Marie Curie-Paris6, UMR-S707, Paris, F-75012, France.
[2]INSERM, UMR-S 707, Paris, F-75012, France.
[3]INRA, UR341 Mathématiques et Informatique Appliquées, Jouy-en-Josas, F-78350.

Model's generalities

The mathematical model is a refinement of that developed by Flahault et al [1], based on the adaptation of the Rvachev and Longini [2] model published by Grais et al [3]. There is a deterministic model in discrete time, including, in the absence of any intervention, the classical four compartments (Susceptible-Exposed-Infectious-Removed; SEIR). It simulates the epidemic at the city level, all the cities being connected by air traffic.

In the previous model formulation [1], the entire city was treated as a homogenous entity without differentiation between age or risk groups. Here, each compartment was divided into sub-groups to take into account heterogeneities in the transmission potential or in the propensity to travel. A second improvement concerns the transmission rate that was considered as having a periodic form. However, since there are very few data available to differentiate transmission rates, for the current analysis we made the assumption of proportional mixing [4] and considered identical individual number of contacts per unit time for all sub-groups. The fraction of contacts resulting in transmission was also taken identical for all sub-groups.

In the absence of any control measure, the principal features of the model are the following:

- the model is a system of difference equations in a discrete time space (time step = 1 day);
- the population is divided, in the absence of any treatment, in four disease states (Susceptible-Exposed-Infectious-Removed) within each city i (i = 1,2, …52). Each of the state variables are divided into k sub-groups (k= 1,…,k_i; here k_i,=K=5 for all cities). These sub-groups may correspond to age classes or different socio-economic categories. The default formulation is to consider these groups as age classes (0-14, 15-24, 25-39, 40-64 and > 64). For each city in the model the age distribution was obtained from the International Population Database (http://www.census.gov). As the age distribution of the population was not available for each city in the model, we assumed that the age distribution of the city was that of the country as a whole.
- the state variables of the model for each city i are:
  - $S_{ik}(t)$: number of susceptible individuals on day t in class k;
  - $E_{ik}(\tau, t)$: number of latent individuals on day t infected τ days ago in class k (here latency is assumed to be equivalent to incubation);
  - $I_{ik}(\tau, t)$: number of infectious individuals on day t infected τ days ago in class k (here all infectious individuals are assumed to be symptomatic);
  - $R_{ik}(t)$: number of recovered (immune) individuals on day t in class k.

To follow the evolution of the disease in each city, two additional state variables are introduced:

- o $W_{ik}(t)$: number of individuals who become infectious on day t (daily incidence) in class k;
- o $B_{ik}(t)$: number of new infectious individuals in class k reported to the health authorities on day t (computed as a fraction of $W_{ik}(t)$);

- for each city i and for each sub-group k, the population size (n_{ik}) is assumed constant over time:

$$S_{ik}(t)+\sum_{\tau=0}^{\tau_1} E_{ik}(\tau,t)+\sum_{\tau=0}^{\tau_2} I_{ik}(\tau,t)+R_{ik}(t)=n_{ik} \quad (1)$$

where τ_1 and τ_2 are the maximum length of the latent and infectious periods respectively.

Since the simulation horizon was relatively short, no natural demographic dynamic was included.

- the infection distributions (probability of being in a given state or transition probability from one state to another) are defined, by the following discrete probability distributions similarly to [2]
  - o $f(\tau)$: the probability for an individual to be in the latent state (τ = 0,1,…, τ_1, $f(0)$=1);
  - o $g(\tau)$: the probability for an individual to be in the infectious state (τ = 0,1,…, τ_2, $g(0)$=0);
  - o $h(\tau)$: the probability for an individual to be in the removed state (τ = 0,1,…, τ_2+1, $h(0)$=0);
  - o $\gamma(\tau)$: the probability that a latent individual becomes infectious on day τ+1 given that he was latent on day τ (τ = 0,1,…, τ_1)
    - $\gamma(\tau) = 1 - f(\tau+1)/ f(\tau)$
  - o $\delta(\tau)$: the probability that an infectious individual recovers on day τ+1 given that he was latent on day τ (τ = 0,1,…, τ_2)
    - $\delta(\tau) = 1 – [f(\tau+1)+ g(\tau+1)- f(\tau)]/ g(\tau)$

  These distributions were fixed at values close to those of Rvachev and Longini [2], calculated to reproduce the 1968 pandemic.
- the infection process is described in each city by a separate but identical set of equations. Some parameters of these equations are equal for all cities; others are specific for each city.
- the global spread of influenza is modelled by a symmetric matrix connecting all the cities, its elements being defined as the daily passenger flow from a city to another. Only susceptible and latent individuals travel (infectious individual do not).
- the model takes into account the seasonal pattern followed by influenza (high winter and low summer incidence in Northern hemisphere) and the delay of approximately 6 months in influenza activity between the Northern and Southern hemispheres. Thus, cities are divided into three zones according to the geographical position: Northern hemisphere, Southern hemisphere and equatorial zone. Following standard formulation for including seasonality in the transmission rate in influenza models [5], we found the function including harmonic terms that fits original estimates of the transmission parameter values cited in Grais et al [3]. The formulation of this function is as follows:

$$\beta(month)=\beta_0 *\left\{1+\beta_1 \cos\left[\frac{2\pi(month+shift)}{12}\right]\right\} \quad (2)$$

We assume that transmission rates are constant over one month. To distinguish the Northern and the Southern hemispheres we took a *shift* of phase in cosine arguments but equal values for β_1. Cities from equatorial zone are not affected by the seasonal trends (β_1=0). β_0 represents the *basic rate of transmission* in the absence of any seasonality character of transmission and β_1 is the *amplitude of seasonal effect*.

Modeling of interventions

Six prevention and control measures are considered in the model. These measures are applied, specifically for each city and for each sub-group inside the city, from a given date or if the number of total reported infectious cases since the beginning of the pandemic is above a predefined threshold.

1. *Antiviral prophylaxis* reduces the transmission rate and the probability corresponding to a change in state (latent -> infectious) accounting for (i) the reduced susceptibility of treated individuals (ii) the reduction of the probability of an infection to be symptomatic (and hence infectious) for a treated individual and (iii) the reduction of the infectiousness of infected individuals previously prophylactically treated. Susceptible individuals treated are given a single course of antivirals. Once the duration of the antiviral prophylaxis is finished, the individuals are assumed to re-enter the untreated susceptible compartment. We assume that all the prophylactically treated individuals continue to receive antivirals (as therapy) if they become infectious.
2. *Masks use* applied to susceptible and latent people, as prophylactic intervention, reduces the transmission rate, illustrating the decrease in the probability of becoming infected for an individual using a mask given contact with an infectious person. Once the use of masks implemented, independently of the use of other prophylaxis interventions, this measure is assumed to be applied during the entire duration of the pandemic.
3. *Vaccination* as prophylactic measure was modeled by a parameter diminishing the number of susceptible individuals. Two policies of administration were considered here. First, vaccination with pre-pandemic influenza vaccines was globally modeled by a coefficient affecting the number of susceptible individuals and representing the global effect of the policy in population. Second, a pandemic vaccination campaign (with vaccine updated for matching pandemic circulating strains) was introduced by taking into account vaccination coverage and vaccine efficacy. During vaccination campaign, the proportion of population to be vaccinated is specified daily.
4. *Limitation of air travel* between cities was modeled by the reduction of the entries of the transportation matrix, specifically for each origin-destination city pair.
5. *Antiviral therapy* diminishes, for infectious treated individuals, the transmission rate (illustrating the reduction of infectiousness of those individuals) and the length of the infectious period and thus the probability of the transition infectious -> recovered.
6. *Isolation* was applied to non-treated and treated infectious individuals but not to latent individuals (who are not symptomatic). Isolated individuals do not spread infection.

In the mathematical formulation, the implementation of these interventions results in the introduction of five supplementary state variables for every sub-group within each city:

- $S^P_{ik}(t,t^P)$: susceptibles under antiviral prophylaxis having started their prophylaxis t^P days ago ;
  - $E^P_{ik}(\tau,t)$: latents under antiviral prophylaxis ;
  - $I^T_{ik}(\tau,t)$: infectious under antiviral treatment ;
  - $I^{Is}_{ik}(\tau,t)$: isolated but non treated infectious individuals;
  - $I^{TIs}_{ik}(\tau,t)$: isolated and treated infectious individuals.

Three additional compartments (V_{ik}, S^M_{ik} and S^{PM}_{ik}) explicitly represented in the Figure 1 of the main text are only implicitly calculated, as part of S_{ik}, S^P_{ik}, E_{ik}, E^P_{ik} dynamics.

As the model includes all six interventions described above, simulations may be performed including all, none or several of the control measures implemented. When a specific measure is not implemented all the corresponding parameters are set to zero.

In the current analysis the parameters related to interventions were equal for all cities.

New infections within each city

The infection process is generated using the standard mass action formulation. The number of newly infected individuals (in latent state) on day *t* in each class *k* of every city *i* is calculated as the product of the number of susceptibles, number of infectious individuals and the transmission rate, β_{ijk} (taken identical for all sub-groups in a city) affected by coefficients modelling the interventions:

$$E_{ik}(0,t) = \left(1 - c^M_{ik} e^M_{ik}\right) \frac{S_{ik}(t)}{n_i} \sum_{j=1}^{K} \sum_{\tau=1}^{\tau_2} \beta_{ijk}(t) \left[I_{ij}(\tau,t) + \left(1 - e^T_{ij}\right) I^T_{ij}(\tau,t) \right] \quad (3)$$

where n_i is the population of city *i*, e^M_{ik} and c^M_{ik} are, respectively, the efficacy in the transmission reduction and the coverage of the masks use, β_{ijk} is the transmission rate for the infectious individuals of group *j* to the susceptibles of group *k* and e^T_{ij} is the treatment efficacy.

Another class of latents, E^P, those coming from susceptibles treated by prophylaxis, is also incremented each day by new infections. Since an infected individual in latent state - here equivalent to incubating - does not exhibit any symptom, he continues to be considered susceptible and hence to be given propylaxis if that was the case before infection.

$$E^P_{ik}(0,t) = \left(1 - c^M_{ik} e^M_{ik}\right) \frac{\left(1 - e^{P1}_{ik}\right)}{n_i} \sum_{t^P=0}^{d^P} S^P_{ik}\left(t,t^P\right) \sum_{j=1}^{K} \sum_{\tau=1}^{\tau_2} \beta_{ijk}(t) \left[I_{ij}(\tau,t) + \left(1 - e^T_{ij}\right) I^T_{ij}(\tau,t) \right]$$

(4)

where e^{P1}_{ik} represents the efficacy of the prophylaxis in reducing the transmission rate (by diminishing the probability of acquiring infection) and d^P is the duration of a prophylactic antiviral treatment.

During the same time step, the number of newly treated susceptibles by prophylaxis is given by:

$$S^P_{ik}(t,0) = c^P_{ik} S_{ik}(t) \quad (5)$$

where c^P_{ik} is the coverage of antiviral prophylaxis.

Travel between cities, transportation operator

The transportation network is quantified by passengers flows between cities: σ_{ilk} represents the average daily passenger from city *i* to city *l* belonging to the class *k*.

Susceptible and latent individuals are assumed to travel proportionally to their fraction in each city. As in [2], a transportation operator is applied to the dynamics of the susceptible and latent persons (equations 6 and 7):

$$\Omega[S_{ik}(t)] = S_{ik}(t) + \sum_{l=1}^{L}\left[\left(1-c_{lk}^{Tr}\right)S_{lk}(t)\frac{\sigma_{lik}}{n_{lk}} - \left(1-c_{ik}^{Tr}\right)S_{ik}(t)\frac{\sigma_{ilk}}{n_{ik}}\right] \quad (6)$$

$$\Omega[E_{ik}(t)] = E_{ik}(t) + \sum_{l=1}^{L}\left[\left(1-c_{lk}^{Tr}\right)E_{lk}(\tau,t)\frac{\sigma_{lik}}{n_{lk}} - \left(1-c_{ik}^{Tr}\right)E_{ik}(\tau,t)\frac{\sigma_{ilk}}{n_{ik}}\right] \quad (7)$$

where L represents the number of cities, here $L = 52$. In both equations c_{ik}^{Tr} and c_{lk}^{Tr} represent the proportions of transportation that is stopped in corresponding cities.

The same operator is applied to the susceptibles and latents treated by prophylaxis (S^P and E^P respectively).

For each pair of cities of the network, the distribution of travellers in sub-groups is assumed to be equal to the mean of the two demographic distributions (those of the origin and de destination cities).

Infection dynamics within and between cities

Modelling the potential natural immunity acquired during previous infections with similar strains, the initial number of susceptible individuals in class k of city i is assumed to be a proportion α_{ik} of the population in this subgroup of the city, n_{ik}. The global potential effect of the pre-pandemic vaccination is integrated by a supplementary parameter v_{ik} affecting the number of susceptible individuals.

$$S_{ik}(0) = \alpha_{ik} v_{ik} n_{ik} \quad (8)$$

The number of susceptibles at time t+1 (equation 9) is obtained from the number of susceptible individuals at time t, taking into account the population migration (via the transport operator Ω, equation 6), from which one withdraws the number of new infections (calculated via the mass action term detailed in equation 3), the number of newly treated susceptibles (equation 5) and the number of vaccinated people and adds the number of previously treated susceptibles who have completed the course of prophylaxis. The coverage and the efficacy of the pandemic vaccination campaign are c_{ik}^{V} and e_{ik}^{V} respectively.

$$S_{ik}(t+1) = \left[1-c_{ik}^{V}(t)e_{ik}^{V}\right]\left\{\Omega\left[S_{ik}(t)\right] + \Omega\left[S_{ik}^{P}(t,d^{P}+1)\right] - E_{ik}(0,t) - S_{ik}^{P}(t,0)\right\} \quad (9)$$

The dynamics of susceptibles treated by prophylaxis is obtained in a similar manner:

$$S_{ik}^{P}(t+1,t^{P}+1) = \Omega\left[S_{ik}^{P}(t,t^{P})\right] - \left(1-c_{ik}^{M}e_{ik}^{M}\right)\frac{\left(1-e_{ik}^{P1}\right)S_{ik}^{P}(t,t^{P})}{n_i}\sum_{j=1}^{K}\sum_{\tau=0}^{\tau_1}\beta_{ijk}(t)\left[I_{ij}(\tau,t) + \left(1-e_{ij}^{T}\right)I_{ij}^{T}(\tau,t)\right]$$

(10)

In the computational algorithm, the initial number of latent individuals is strictly positive for the city representing the pandemic source only and is zero for all other cities.

The dynamic of the latent individuals receiving or not antivirals as prophylaxis is given by equations (11) and (12):

$$E_{ik}(\tau+1,t+1) = \left[1-\gamma_{ik}(\tau)\right]\Omega\left[E_{ik}(\tau,t)\right] \qquad \tau = 0,1,\ldots,\tau_1 - 1 \quad (11)$$

$$E_{ik}^{P}(\tau+1,t+1) = \left[1-\gamma_{ik}(\tau)\right]\Omega\left[E_{ik}^{P}(\tau,t)\right] \qquad \tau = 0,1,\ldots,\tau_1 - 1 \quad (12).$$

The next equations describe the dynamics of non-treated (equation 13), treated (equation 14), isolated non treated (equation 15) and isolated treated (equation 16) infectious individuals; two expressions are given, according to the time of infection. For the non-isolated non-treated or treated infectious compartments, if the delay from infection is less than the

maximum of latent period, τ_1, the number of infectious persons at time t+1 infected τ+1 days ago is given by the number of latents at the previous time step (via their transportation operator Ω) who become infectious plus the number of infectious persons at the previous time step who remain infectious and minus those who are isolated. If the delay from infection is between the maximum length of the latent period, τ_1, and the maximum length of the infectious period, τ_2, the equation only includes the infectious individuals at the previous time that did not recover minus those who are isolated. The initial number of infectious individuals is zero for all cities.

$$I_{ik}(\tau+1,t+1)=\begin{cases}\left(1-c_{ik}^{T}\right)\gamma_{ik}(\tau)\Omega\left[E_{ik}(\tau,t)\right]+\left(1-e_{ik}^{Is}c_{ik}^{Is}\right)\left[1-\delta_{ik}(\tau)\right]I_{ik}(\tau,t) & \tau=0,1,...,\tau_1\\ \left(1-e_{ik}^{Is}c_{ik}^{Is}\right)\left[1-\delta_{ik}(\tau)\right]I_{ik}(\tau,t) & \tau=\tau_1+1,...,\tau_2-1\end{cases} \quad (13)$$

$$I_{ik}^{T}(\tau+1,t+1)=\begin{cases}\gamma_{ik}(\tau)\left\{c_{ik}^{T}\Omega\left[E_{ik}(\tau,t)\right]+\left(1-e_{ik}^{P2}\right)\Omega\left[E_{ik}^{P}(\tau,t)\right]\right\}+\left(1-e_{ik}^{Is}c_{ik}^{Is}\right)\left[1-\delta_{ik}^{T}(\tau)\right]I_{ik}^{T}(\tau,t) & \tau=0,1,...,\tau_1\\ \left(1-e_{ik}^{Is}c_{ik}^{Is}\right)\left[1-\delta_{ik}^{T}(\tau)\right]I_{ik}^{T}(\tau,t) & \tau=\tau_1+1,...,\tau_2-1\end{cases} \quad (14)$$

$$I_{ik}^{Is}(\tau+1,t+1)=\left[1-\delta_{ik}(\tau)\right]\left[e_{ik}^{Is}c_{ik}^{Is}I_{ik}(\tau,t)+I_{ik}^{Is}(\tau,t)\right] \qquad \tau=0,1,...,\tau_2-1 \quad (15)$$

$$I_{ik}^{TIs}(\tau+1,t+1)=\left[1-\delta_{ik}^{T}(\tau)\right]\left[e_{ik}^{Is}c_{ik}^{Is}I_{ik}^{T}(\tau,t)+I_{ik}^{TIs}(\tau,t)\right] \qquad \tau=0,1,...,\tau_2-1 \quad (16)$$

In the four equations above c_{ik}^{Is} is the proportion of isolated individuals and e_{ik}^{Is} is the efficacy of isolation. In equation (14) e_{ik}^{P2} represents the efficacy of the prophylaxis on the probability of illness given infection. In equations 14 and 16, transition probability I->R is different for individuals receiving treatment (δ_{ik}^{T}) from that of those untreated, as a consequence of an assumed shorter infectious period (by one day) under therapy.

The incidence on day t+1 (equation 17) is calculated as sum of all new infectious (viewed as the product of the transportation operator applied to the latent individuals on day t and the transition probability from the latent to the infectious state). The daily reported incidence is simply a fraction of the daily incidence, ρ_{ik} denoting the reporting rate (equation 18).

$$W_{ik}(t+1)=\sum_{\tau=0}^{\tau_1}\gamma_{ik}(\tau)\left\{\Omega\left[E_{ik}(\tau,t)\right]+\left[1-e_{ik}^{P2}\right]\Omega\left[E_{ik}^{P}(\tau,t)\right]\right\} \quad (17)$$

$$B_{ik}(t+1)=\rho_{ik}W_{ik}(t+1) \quad (18)$$

References

1. Flahault A, Vergu E, Coudeville L, Grais, R (2006) *Strategies for containing a global influenza pandemic.* Vaccine **24**: 6751-6755.
2. Rvachev L, Longini IM, Jr (1985) A mathematical Model for the global spread of influenza. Mathematical Biosciences 75: 3-22.
3. Grais RF, Ellis JH, Glass GE (2003) Assessing the impact of airline travel on the geographic spread of pandemic influenza. Eur J Epidemiol 18: 1065-1072.
4. Jacquez JA, Simon CP, Koopman J, Sattenspiel L, Perry T (1988) Modeling and analyzing HIV transmission: the effect of contact patterns. Math Biosci 92: 119-199.
5. Keeling MJ, Rohani P, Grenfell BT (2001) Seasonally forced disease dynamics explored as switching between attractors. Physica D 148: 317-335.
